# Book Reviews

**Published:** 1912-08

**Authors:** 


### BOOK REVIEWS

PROGRESSIVE MEDICINE. A quarterly digest of advances, discoveries and improvements in the medical and surgical sciences. Edited by Hobart A. Hare, M. D. Assisted by Leighton F. Appleman, M. D. Volume II, June, 1912. Lea & Febiger, Philadelphia.

Hernia is reviewed by W. B. Coley, M. D.; Surgery of the Abdomen, exclusive of hernia; Gynecology, by J. G. Clark,

M. D.; Diseases of the blood, etc., by A. Stengel, M. D., and Ophthalmology by E. Jackson, M. D. The subjects are all well covered.

---

CYCLOPEDIA OF AMERICAN MEDICAL BIOGRAPHY. By Howard A. Kelly, M. D., Professor of Gynecologic Surgery at Johns Hopkins University, Baltimore. Two octavo volumes averaging 525 pages each, with portraits. Philadelphia and London: W. B. Saunders Company, 1912. Per set: Cloth, $10.00 net; Half Morocco, $13.00 net.

Kelly has given a brief outline of the life of every "medical worthy" (distinguished) who has lived in the United States and Canada. While much valuable information is contained in its pages, of course, as could easily have been guessed, a considerable number of distinguished men have not been mentioned. This in no way detracts from the facts given, but leaves some notable additions to be made in the second edition.

---

HOME NURSE'S HANDBOOK OF PRACTICAL NURSING. A Manual for use in Home Nursing Classes, in Young Women's Christian Associations, in Schools for Girls and Young Women, and a working text-book for mothers, "practical" nurses, trained attendants, and all who have the responsibility of the home care of the sick. By Charlotte A. Aikens, Author of "Hospital Management," "Hospital Training-School Methods," "Primary Studies for Nurses," "Clinical Studies for Nurses." 12mo of 276 pages, illustrated. Philadelphia and London: W. B. Saunders Company, 1912. Cloth, $1.50 net.

This valuable little volume deals with home nursing as distinct from the more elaborate technic of hospital practice, and is intended for one trying to become a useful helper to the physician in the home sick-room.

RECENT METHODS IN THE DIAGNOSIS AND TREATMENT OF SYPHILIS. (The Wassermann Reaction and Ehrlich's Salvarsan, "606.") By C. H. Browning, M. D., Lecturer on Bacteriology in the University of Glasgow, and Ivy McKenzie, M. D., Director, Western Asylums' Research Institute, Gasgow. Octavo, 303 pages. Cloth, $2.50, net. Lea & Febiger, Publishers, Philadelphia and New York, 1912.

In this age of research, one discovery treads so rapidly on the heels of another that their wonder and significance are apt to be overlooked. Ehrlich's final success after scientifically testing 606 chemical compounds is amazing in the vista it opens of the conquest of the most widespread and important of all human diseases. Scarcely less remarkable is the timeliness of the discovery of Wassermann's unerring test for the disease which detects it even before the stage of visible lesions has been reached. These two epoch-making discoveries complete each other by enabling the physician to prove the presence or absence of syphilis by Wassermann's test, to treat it radically, if present, with Ehrlich's Salvarsan, and afterwards to demonstrate absolutely when it has been eradicated by reapplying the Wassermann test. In this authoritive work readers will find a full explanation of the theory and principles of Ehrlich's and Wassermann's methods, their application, a discussion of Drs. Browning and McKenzie's cases, which are ample to give the stamp of experience to their writings. The widespread interest in the subject and in the book is shown by the fact that about half the edition was sold within a few days after publication.

ARTERIOSCLEROSIS: Etiology, Pathology, Diagnosis, Prognosis, Prophylaxis and Treatment, with a special chapter on blood pressure. By Louis M. Warfield, A. B., M. D., with an introduction by W. S. Thayer, M. D. Illustrated with 28 engravings. C. V. Mosby Co., St. Louis, Mo.

This monograph is a readable and authoritive book on an important subject closely related to modern civilization.

